# Exploring key determinants of health among individuals with serious mental Illness: qualitative insights from a first episode psychosis cohort, 20 years postdiagnosis

**DOI:** 10.1186/s12888-023-05270-1

**Published:** 2023-10-26

**Authors:** Jorunn Nærland Skjærpe, Wenche ten Velden Hegelstad, Inge Joa, Marianne Storm

**Affiliations:** 1 Department of Public Health, Faculty of Health Sciences, University of Stavanger, Stavanger, Norway; 2 TIPS Centre for Clinical Research in Psychosis, Stavanger University Hospital, Stavanger, Norway; 3 Department of Social Studies, Faculty of Social Sciences, University of Stavanger, Stavanger, Norway; 4 Faculty of Health Sciences and Social Care, Molde University College, Molde, Norway; 5 Research Department, Research Group of Nursing and Health Sciences, Stavanger University Hospital, Stavanger, Norway

**Keywords:** Qualitative design, Individual interviews, Service users, Mental disorder, Psychosis, Early intervention, Mental healthcare

## Abstract

**Background:**

Individuals with serious mental illness (SMI) are more likely to experience functional decline, low well-being, comorbidities, shorter lifespan, and diminished quality of life than the general population. This qualitative study explores determinants of health that individuals with SMI perceive as important to their health, well-being, and ability to live a meaningful life.

**Method:**

We conducted interviews with 13 individuals with early detected first episode psychosis as part of a 20-year follow-up study of a larger cohort. Interview data were analyzed using qualitative content analysis.

**Results:**

Analysis identified two themes comprising eight categories representing determinants of health. The first theme reflected management of mental and physical health. Categories in this theme were: access to mental healthcare adapted to individual needs, strategies during deterioration, use of psychotropic medication, maintenance of physical health and lifestyle. The second theme reflected social health determinants in coping with mental illness and comprised three categories: family and friends, engaging in meaningful hobbies and activities, and the influence of employment on mental health.

**Conclusions:**

Individuals with SMI outlined mental, physical, and social determinants of health that were important for their health, well-being, and ability to live a meaningful life. In future clinical practice, coordinated care addressing the complexity of health determinants will be important.

## Background

Determinants of health are factors with a significant positive or negative influence on health and well-being [1, 2]. In recent years, there has been growing interest in the determinants of serious mental illness (SMI) due to its association with increased risk of functional decline, comorbidities, and premature death [3]. SMI, according to DSM-IV criteria, encompasses schizophrenia, schizophreniform disorder, schizoaffective disorder, brief psychotic disorder, delusional disorder, severe mood disorders, or psychosis not otherwise specified [4]. About 1-3.5% of the Norwegian population will fulfill lifetime criteria for an SMI disorder [5]. First onset of a psychotic disorder typically occurs in late adolescence or early adulthood, leading to impairments that can affect social, educational, and vocational development and physical health [6]. In addition, the risk of suicide is substantial [7, 8]. Overall, individuals with SMI often experience diminished well-being, social functioning, quality of life [9], and lifespan compared to the general population [10]. Worryingly, these gaps have grown in recent decades, likely due to social rather than biological factors [11].

Huggard et al. [12] provide an overview of the determinants of mental illness (i.e., community environments, social interaction and support, life experiences, lifestyles, financial factors, education, employment, housing, living conditions such as living alone or with family, and marital status). Social interaction and support may help individuals with SMI to contribute to their communities and recover from mental illness [13]. Spending time with family, friends, and colleagues can build confidence and promote a positive self-image [14]. In contrast, difficult life experiences such as the death of family members, caregiving burdens, conflicts, or dysfunctional family life can negatively influence mental health [12]. Employment can substantially impact an individual’s well-being by providing social arenas and a sense of belonging [15]. However, individuals with SMI often struggle to establish and maintain social relationships and employment [13] and commonly lack social interaction and support [15–17].

Access to healthcare is a key health determinant [18], vital in alleviating symptoms, promoting health, supporting activities of daily living, and enhancing the well-being of individuals with SMI [19–21]. A study [22] of professionals and patients found that insufficient healthcare can result from patient-related factors such as lack of treatment motivation or non-attendance of consultations. Stigmatizing attitudes among professionals and low optimism regarding improved mental health can reduce willingness to seek healthcare [12]. Mental health stigma can be more harmful than the illness itself as it can lead to social exclusion and inadequate healthcare [23]. For those who do seek healthcare, non-adherence to complex medication regimens is an additional problem [24]. Insufficient healthcare can also stem from systemic factors such as diagnostic overshadowing, poor availability of general practitioners (GPs) [22], an absence of integrated care models for mental and physical comorbidity, and insufficient development of community-based services [25]. These issues can limit access to healthcare and impede proper care for individuals with SMI [25–27]. Access to and utilization of physical primary and specialist healthcare is a pragmatic and cost-effective approach to improving health equity in individuals with SMI, as poor physical health outcomes are attributed to inadequate health prevention, unfavorable health behavior, and reduced life expectancy [28].

A recent scoping review [21] emphasized that proper healthcare for individuals with SMI necessitates appropriate coordination of care to address multiple health aspects. Such coordination has shown promise in overcoming the impact of negative health determinants [18]. The US Agency for Healthcare Research and Quality (AHRQ) refers to care coordination as “the deliberate organization of patient care activities between two or more participants (including the patient) involved in a patient’s care to facilitate the appropriate delivery of healthcare services” [29, p. 6]. Coordinated care is continuous and delivered by multiple professionals who effectively communicate and collaborate within and between services [30].﻿ Such care might involve integrating care from specialist and municipal services as well as from other professionals to address each individual’s needs [19, 31]. Traditional forms of healthcare may not fully address the social needs of individuals with SMI, which could be hampered by difficulties with self-care, substance and alcohol use, and social problems [15]. Leijten [30] recommends a holistic approach to care coordination that addresses the mental, physical, and social factors that impact health and well-being.

Research has shown that the early course of SMI is the period of the illness where most of the disabling consequences occur [32]. Longer duration of untreated psychosis is associated with worsened prognosis [33]. The main objectives of early intervention should be to prevent or delay the onset of manifest psychosis, reduce the severity of the illness, and minimize negative social consequences [34]. The Scandinavian early intervention in first-episode psychosis study, TIPS (Treatment and Intervention in Psychosis; 1997–2000), was a prospective longitudinal study on the early detection of first-onset psychosis [35]. The study comprised a treatment program for individuals with SMI that centered on easy access to coordinated healthcare to reduce the duration of untreated psychosis and investigate the effects of this reduction. When patients first came into contact with specialist health services, trained professionals assessed them and immediately assigned them to the treatment program [35]. Patients were followed up after one, two, five, and ten years to monitor their progress [35–38]. A 20-year follow-up is now in progress.

Findings from the TIPS study showed that reducing the duration of untreated psychosis was possible [34–39]. Even more promising was the finding that individuals whose illness was detected early consistently showed benefits regarding clinical status and functional outcomes at all follow-up points [34–39]. Early compared to usual detection confers several important advantages: lower likelihood of discontinuing medical treatment, lower chance of relapse, fewer psychiatric admissions, reduced overall symptom severity, improved quality of life, better employment and educational outcomes, higher rates of recovery, better insight, and improved social functioning [40]. Nevertheless, up to 64% of individuals with an early diagnosis of schizophrenia still received mental healthcare 30 years after first contact, even after comprehensive treatment efforts [41]. Identifying the health determinants of individuals with SMI would give further insight into this statistic, and may yield substantial benefits for patients, their families, and the community. In the current study, we aim to qualitatively explore determinants of health among a subgroup of individuals from the TIPS cohort. We ask the following research question: What determinants of health do individuals with SMI perceive as important to their health, well-being, and ability to live a meaningful life?

## Method

### Study setting: the Norwegian healthcare system

Norway’s healthcare system is publicly funded and founded on the principle of universal access for all citizens. The system comprises municipal health and care services and specialist health services that are governed by separate public funding, laws, and regulations. Municipalities are accountable for ensuring citizens have access to primary mental and physical healthcare. This encompasses emergency rooms, housing with professional support, home-based healthcare, and various other social, psychosocial, and medical habilitation and rehabilitation services [42].

Citizens registered in the National Population Register are entitled to the services of a GP. GPs provide treatment for acute and chronic illnesses and preventive care across all age groups and act as gatekeepers to specialist health services, managing healthcare needs beyond the municipality’s scope of competence [42]. The state is responsible for providing specialist health services [43]. For individuals with SMI, these services comprise inpatient and outpatient treatment and care provided by hospitals and community mental health centers (CMHCs), with the latter generally offering a lower level of care than psychiatric hospitals. Individuals with SMI may require both municipal and specialist health services to meet their healthcare needs and prevent deterioration of their health [44].

### The TIPS study

The TIPS study was conducted in specialist health services in four Scandinavian healthcare sectors with similar sociodemographic characteristics [35]. Using a parallel control design, two of these sectors conducted treatment programs for early detection of psychosis, while the other did not. The two sectors that employed early detection were in Rogaland County, Norway, with an approximate population of 370,000. The early detection program comprised information campaigns about psychosis and treatment directed to the general population through newspaper, radio, and cinema advertisements as well as being provided to GPs, social workers, and high school healthcare professionals. Specialized low-threshold early detection teams could be reached via telephone by patients, family, and friends [35]. Patients were treated over two years using a standard treatment protocol [37]. The treatment program consisted of defined antipsychotic medication treatment algorithms [45], multi-family psychoeducation groups [46], and weekly individual psychotherapy [36]. After two years, patients received treatment in standard clinical settings based on national guidelines [47].

### Design, participants, and recruitment

This study has a qualitative exploratory design [48] based on interviews conducted with individuals with SMI [49]. The sample was recruited from the TIPS cohort (*N* = 281) [35, 38], which consists of individuals with SMI from areas employing an early detection program. The TIPS cohort was recruited during the period 1997–2000. Inclusion criteria were that participants were experiencing first-episode schizophrenia, schizophreniform disorder, or schizoaffective disorder (core schizophrenia), delusional disorder, mood disorder with mood-incongruent psychotic features, brief psychotic disorder, or psychosis not otherwise specified, living in one of the participating sites, being 18–65 years old, and within the normal range of intellectual functioning (WAIS-R-based IQ estimate > 70). Inclusion criteria are described in more detail by Melle et al. [38].

Recruitment for this study took place during the initial phase of the TIPS 20-year follow-up (2021–2022). At that time, twenty participants had been contacted, all of whom were invited to participate in the qualitative interviews. The hospital staff responsible for the TIPS 20-year follow-up study obtained written consent from participants, which granted our study’s first author (JNS) permission to initiate contact with them regarding the current study. JNS then contacted potential interviewees via telephone to provide more information about the study. All individuals (*N* = 13) who expressed willingness and provided written consent to participate were included. Reasons for not agreeing included lack of time or too long travel distance.

These thirteen were the participants we were able to access during the available recruitment time and represent approximately 20% of the total TIPS 20-year follow-up sample (*N* = 70). Polit and Beck [50] argue that for qualitative research, ten or fewer interviewees can provide a sufficient amount of data. Our sample comprised five females and eight males. At the time of the interview, all were living independently in the community. The average age at inclusion was 49.5 years (range 38–65 years). All participants had been involved with the TIPS study for approximately 20 years. Table 1 shows the characteristics of the study participants.

**Table 1:** Study participants characteristics

Study participants characteristics		N
Gender	Female	5
	Male	8
Age	38–49	8
	50–59	2
	60–65	3
Employment	Employed	8
	Unemployed	5
Living condition	Living alone	5
	Living with family	8

### Data collection

We used an interview guide [49] (Table 2) that covered thematic areas from the care coordination framework produced by the AHRQ [29]. The thematic areas were as follows: care organization, individual healthcare needs, communication with professionals, involvement in decision-making, care transitions, medication management, self-management, and connections to community resources. These coordination areas can be connected to determinants of health [12–18]. At the end of each interview, participants were invited to provide any further relevant information to address topics not sufficiently covered.

To ensure that the interviews were grounded in the perspectives of individuals with SMI, we solicited feedback on the interview guide from peers with a lived experience of mental illness and recovery. These constituted a group of four co-researchers affiliated with the TIPS program who met regularly with the TIPS group to provide input on research questions, designs, interpretations, and dissemination of research. They all had a history of SMI (three schizophrenia and one schizoaffective disorder). All had experienced psychiatric hospital admission. They were in remission and used medications. They had completed the TIPS-organised course in basic research methods and had volunteered to participate in the TIPS research group.

JNS and WtVH held an online meeting with three members of the co-researcher group in which we presented the preliminary interview guide. Based on feedback, we incorporated open-ended questions after each area, such as “Tell us about your experience with this,“ “How do you perceive the functionality of this?“ and “What are your thoughts on your follow-up?“.

**Table 2:** Interview guide

Interview guide
Care organization What healthcare services are you receiving? What assistance do you receive from professionals to establish contact with the relevant services? What role does your GP have? Could you please share your experience with your services?
Communication and involvement in decision-making What information do you receive about your services? How are you involved in the selection of services? How do professionals consider your preferences and healthcare needs? Do you participate in meetings with professionals within and between services? Can you tell us about your experience with this?
Care transitions How are transitions from inpatient care to home organized? What information is provided to you during transfers? How are your healthcare needs assessed during transfers? How do you perceive the transitions are working for you?
Follow-up of individual healthcare needs How is your health monitored? How do the services you receive meet your needs? What are your thoughts on your follow-up?
Medication management Are you taking medications? What medications are you using? How is your medication use monitored? How do you perceive the effectiveness of this?
Self-management How do you take care of your health? What do you do to make you feel well? What is important for you to have a good life?
Connections to community resources. How do you spend your days? Do you have access to daily activities? How do you perceive the functionality of this? Would you like to add anything?

Interviews were conducted between May 2021 and August 2022 by JNS, who talked with participants in person at the university (*N* = 3), in their homes (*N* = 6), or over the phone (*N* = 4), depending on their preference. Interviews ranged in duration from 20 to 60 min, with an average of 30 min. All interviews were conducted in Norwegian, audio-recorded, transcribed verbatim by a hospital secretary, and translated into English by JNS.

### Data analysis

We performed a qualitative content analysis of the interview data [51–53]. Initially, all authors (JNS, WtVH, IJ, and MS) read the interview transcriptions to comprehensively understand the content. Next, we (JNS, WtVH, IJ, and MS) identified units of meaning, such as words, sentences, and paragraphs pertinent to our research question. These units were condensed by shortening the text while preserving the meaning and were labeled with a code to describe its contents using the NVivo (Version R1) software tool [54]. We (JNS, WtVH, IJ, and MS) then grouped similar codes and synthesized and abstracted the text into eight categories representing determinants of health. These categories constituted a descriptive level of content, reflecting the text’s manifest content. We (JNS and MS) connected categories with related determinants of health and abstracted these into two themes on an interpretative level. These themes expressed the text’s latent content through a thread of underlying meaning throughout the meaning units, codes, and categories.

Although we describe this process chronologically, our actual process continually moved between the original text transcriptions and the various analysis phases before all authors agreed on a final set of categories and themes that aligned with the study’s aim and research question [53]. Each of the study’s four authors, all from different professions (social educator, clinical psychologist, psychiatric nurse, and nurse), contributed to the analysis to enhance the study’s trustworthiness [51]. Table 3 displays selected examples from the analytic procedure.

**Table 3:** Examples from the analytic procedure

Examples from the analytic procedure				
Meaning unit	Condensed meaning unit	Code	Category	Theme
I have a therapist. I have had this therapist for 25 years. I am fortunate in that. The good thing about having the same therapist is that she knows everything. There is no need to repeat myself. What is done is done. I am so grateful that she can handle me and I can be myself.	I have had the same therapist for 25 years. Having a therapist who knows my history and can tolerate me is so good.	Continuity in relationships	Access to mental healthcare adapted to individual needs	Managing mental illness and physical health problems
I lost my wife about a year ago. We used to travel together to several countries. Yes, we have seen a lot and experienced a lot. Had a good time. We had many − 30 years together. So, I must be content with that.	I lost my wife a year ago. We were married for 30 years. I fondly reminisced about our shared adventures.	Loss of family	Family and friends – a double-edged sword	Social health determinants in coping with mental illness

### Ethical considerations

The study received ethical approval from the Regional Committee for Medical and Health Research Ethics (REK), Southeast Health Region. It was also approved by the Data Protection Officer at Oslo University Hospital and Stavanger University Hospital. These approvals ensure that the research project adheres to ethical guidelines and regulations and provides the approval for information security and privacy services. The study adhered to the principles outlined in the Helsinki Declaration and the research guidelines from the Stavanger University Hospital. The participants were recruited voluntarily and informed about the study’s confidentiality, that participation was voluntary, and that they had the right to withdraw anytime. Before the interviews, written informed consent was obtained from all participants. The participants signed informed consent, which indicated that they understood the study’s aim and the nature of participation and allowed the interviews to be audio recorded. The names of interviewees were replaced by numbers (Participants 1–13).

## Results

Below, we present the two themes and eight categories representing the determinants of health described as important by participants to their health, well-being, and ability to live a meaningful life. Themes and categories are illustrated in Table 4.

**Table 4:** Themes and categories

Themes	Categories
1: Managing mental illness and physical health problems	1: Access to mental healthcare adapted to individual needs 2: “Living with mental illness is not easy” - strategies during deterioration 3: “I must take my medication to stay well “- use of psychotropic medication 4: Maintenance of physical health “challenged by misinterpreted symptoms” 5: Lifestyle
2: Social health determinants in coping with mental illness	6: Family and friends – a double-edged sword 7: Engaging in meaningful hobbies and activities 8: “Having a job has probably saved me” – the influence of employment on mental health

### Theme 1: Managing mental Illness and physical health problems

The five categories within this theme were: access to mental healthcare adapted to individual needs, strategies during deterioration, use of psychotropic medication, maintenance of physical health, and lifestyle.

#### Category 1: Access to mental healthcare adapted to individual needs

The interviewees’ current mental health status varied. Some had been without symptoms for years, others experienced intermittent symptoms, and a number had continuous symptoms to varying degrees. A majority of interviewees expressed satisfaction with mental healthcare services, but others wished for more involvement in the decision-making process regarding their follow-up care. As one stated:

*I wish professionals were more interested in my needs and preferences. They have not usually inquired about my desired healthcare or follow-up plan. There have been no alternatives besides seeing a psychologist* (Participant 11, female).

Approximately half of the participants interviewed primarily relied on their GP for managing their mental health, while the remaining group received additional support through municipal mental healthcare and/or outpatient treatment from the CMHC. Several participants regularly saw mental health professionals from the municipality in professionals’ offices or at home and discussed the challenges faced trying to organize the necessary support. One individual had medication delivered twice weekly and had a pill dispenser filled weekly by home-based healthcare services. He also underwent drug substitution therapy for substance dependence (LAR). Another participant received monthly depot injections from professionals at the CMHC, while also receiving visits from municipal mental health professionals every other week for consultations and seeing a CMHC psychiatrist every three to four months.

Several participants received outpatient treatment from the CMHC, with varying experiences. Some were satisfied while others found it to be of little use. One person noted that the advice he received was mostly familiar to him, such as making sure to visit others and stay physically active. Another stated:

*The psychologist treated me like I was sick. It was fine at first, but then I felt like she was making me sicker than I actually was. I know that when I am healthy, I am healthy, so I would like to concentrate on that* (Participant 2, female).

This person now received home visits from a municipal mental health professional every other week. She said that she could talk to this worker about anything and that she was happy with this follow-up. One woman, who had had the same therapist for 25 years, emphasized the importance of continuity in the recovery processes, having a therapist who knew her history and could tolerate her as a person, even during disagreements. She stated:

*I changed therapists so often when I was younger. It did not work for me. It was like digging and digging, the same thing always. I just got sick of it. The benefit of having the same therapist is that she knows everything. There is no point in repeating it. Even when I was admitted - she came there too. I have told her I am so glad she puts up with me* (Participant 3, female).

#### Category 2: “Living with mental Illness is not easy” - strategies during deterioration

Close follow-up was beneficial during times of deteriorated or unstable mental health. Some participants expressed frustration with their vulnerability to deterioration and their difficulty managing symptoms, particularly when faced with multiple stressors. One man expressed:

*It is frustrating that I cannot handle more. I cannot focus on one thing at a time whenever I become unwell. There is simply too much going on, and it becomes overwhelming. I find myself getting caught up in trivialities. However, I must accept that this is part of my life. Living with mental illness is not easy* (Participant 1, male).

Interviewees talked about their struggle to recognize symptoms and distinguish reality from perception during periods of deteriorated mental health. One could sense unstable mental health but found it difficult to respond to symptoms:

*During episodes of mania, I often observe a rapid deterioration. However, recognizing the severity of my condition becomes difficult, despite being aware of my declining well-being. Managing my unstable mental health becomes challenging, necessitating assistance seeking mental healthcare and receiving proper treatment from professionals* (Participant 10, female).

Some interviewees had developed emergency psychiatric care plans with the help of professionals and family members. These plans included information on what to do in case of deterioration and how to identify warning signs. One man detected changes in sleep quality when he began to experience deterioration. He explained:

*Things start to go wrong when I lose sleep. I believe my lack of sleep is a symptom of deterioration rather than a cause. I occasionally use zopiclone to help me fall asleep and I have previously been prescribed diazepam during a period of high stress and anxiety related to work. It allowed me to unwind in the evenings and improved my sleep and mental health* (Participant 9, male).

One interviewee received a follow-up during a period of deterioration where CMHC professionals came to her home for conversations and walks. She also received medication delivery from home-based healthcare twice daily until her mental health was stable. Some participants had turned to the CMHC’s acute team for care during periods of deterioration and were satisfied with the follow-up, which included home visits. One participant shared that during a difficult period, he received follow-up conversations with a psychiatric nurse in the emergency room. He stated:

*If you feel like your life is truly bad, you can have a couple of consultations there - then at least you get the worst out* (Participant 7, male).

The participant expressed a desire to avoid psychiatric admission. However, during periods of deteriorated mental health, such care might be necessary to stabilize severe symptoms. Many participants experienced multiple psychiatric admissions. They reported that they were usually admitted once they requested it, and their GP typically referred them. Some shared that they recognized when hospitalization was necessary. One said:

*At times, I have asked to be taken to the hospital, and once I am there, everything falls apart, and I end up experiencing a full-blown psychosis* (Participant 2, female).

Another person explained:

*When I become ill, it becomes pretty severe, particularly during manic episodes. Mania is extremely challenging, and being in the hospital is my best option. Retaining and shielding myself from everything is comforting to calm down* (Participant 10, female).

#### Category 3: “I must take my medication to stay well “- use of psychotropic medication

Multiple participants stated that their mental health had improved with age. They highlighted psychotropic medication as the primary reason for stable mental health. One woman said:

*I confided in a friend of mine who is a psychiatric nurse that there is one thing I fear the most: getting sick again. This thought stresses me out, and stress can trigger my illness. My friends, family, and professionals have all warned me that stopping my medication could lead to deterioration, which I have also learned from previous experiences. I must take my medication to stay well* (Participant 2, female).

For some individuals, the desire to distance themselves from their previous experiences with SMI was central, as excessive dwelling on these experiences negatively affected their well-being. Despite this, they acknowledged the necessity of adhering to a daily medication routine, noting that this constantly reminded them of their previous condition. They recognized that lifelong medication intake was likely necessary. As one expressed it:

*I rely on medications to maintain optimal health. Some people try to reduce their medication, but it is not something I want to try. The medication protects me from stress and helps with sleep, which is crucial, especially since my workdays can be stressful* (Participant 10, female).

The interviewees say their GPs follow up on medications and write prescriptions. Several talked about how they had spent years finding the best type of medication for their symptoms and the optimal dosage with the desired effect. One person said:

*I have previously been given excessively high doses, which made me feel sluggish and overmedicated. Despite trying various medications, none had the desired effect and I just got worse. I have quit all medications several times and started over again to find the proper medication, often during hospitalization. This has been beneficial in order to be prescribed proper medication, which has made it easier to manage my medication regimen* (Participant 10, female).

Some interviewees had experienced unwanted side effects such as fatigue, seizures, emotional flattening, and weight gain. One man described becoming mentally ill and hospitalized for the first time in high school. He was prescribed olanzapine, which made it challenging to complete his education. His mind was slower, learning was difficult, and he received poor grades despite studying a lot, eventually opting to end his education. Another said:

*I think the medication does something to my metabolism. I do not eat more than before but still gain weight* (Participant 9, male).

One interviewee shared that he was prescribed antipsychotic medication several times when he experienced poor mental health. Despite this, he stopped taking medication due to side effects, even when advised against this by professionals. While some interviewees said that they could taper off medication with the help of their GP, others had not yet found a medication that made them symptom-free. One man said:

*I have tried several types of antipsychotic medication but still hear voices. I also tried without medication but quickly became ill again. It only lasted a few months, and then it got completely messed up in my head. I eventually realized the importance of medication and now take it regularly. The ones I use now are the ones that have had the best effect* (Participant 13, male).

Numerous participants who discontinued their medication reported a subsequent deterioration of their mental health. This led to an exacerbation of symptoms and substantially impacted their well-being. Moreover, it often resulted in the need for sick leave, hospitalization, and an increased reliance on coordinated healthcare. One participant said:

*I stopped taking my medication when I was about to become a father. I thought I would try without it. However, I lost sleep, became ill, and was hospitalized for a month. I was last hospitalized five years ago when I also stopped taking medication. During that hospitalization, I started on antipsychotics again and have been stable since then* (Participant 9, male).

#### Category 4: Maintenance of physical health “challenged by misinterpreted symptoms”

Several interviewees had experienced physical health problems that affected their well-being and ability to carry out everyday tasks. Some described it as easier to manage physical health challenges than mental ones, as physical challenges were more concrete. As one stated:

*I have a herniated disc in my back and a sore shoulder that can cause discomfort and affect my sleep and ability to work. However, it is nothing compared to my experienced mental chaos* (Participant 12, male).

Participants said that it was common for their GPs to perform routine health checks, follow up on physical health, and coordinate with specialist health services as needed.

Overall, they were satisfied with the follow-up regarding their physical health. The conditions mentioned included cardiovascular diseases, cancer, kidney diseases, metabolic diseases, uric acids, gastrointestinal diseases, tinnitus, and different types of pain, including back pain, arm pain, shoulder pain, joint pain, fibromyalgia, and headaches. Interviewees were eager to manage these problems through, for instance, medical appointments, medication, exercise, and physical therapy.

There were instances where the interviewees talked about where doctors erroneously interpreted their psychical symptoms as part of a psychiatric condition. In the research literature, this is referred to as “diagnostic overshadowing” [22, 28]. One such instance involved a woman who had a kidney infection, which was mistakenly recognized as a psychiatric issue. She also reported being prescribed anxiety medication for pneumonia as her doctors believed it to be anxiety-related. It was also discussed that metabolic diseases could have contributed to mental illness. For example, one woman received radioiodine treatment for hyperthyroidism and had stable thyroid function. She suspected that her previous hyperthyroidism might have triggered episodes of mania as she now experiences stable mental health. There were also mentions of lifestyle-related issues such as inactivity, smoking, and obesity causing physical health problems. One stated:

*As I have gotten older, I have noticed a significant decrease in my energy levels and motivation. I have been dealing with many physical health problems, including high blood pressure, gout, and high cholesterol, all requiring medication. My smoking habit may also impact my health, although I have been fortunate not to experience substantial breathing difficulties thus far* (Participant 13, male).

#### Category 5: Lifestyle

Some interviewees noted the importance of eating nutritious food to maintain a healthy lifestyle and normal weight. One interviewee decided to stop smoking tobacco and experienced a notable decrease in the severity of psychotic symptoms. In addition, several individuals were aware of the importance of keeping alcohol consumption moderate, not drinking to feel better, and staying away from illicit drugs. One said:

*I have never used anything other than alcohol. Doctors and psychologists have told me that if I start using illicit drugs, the chance of psychosis increases considerably. So just abstain from it, they said- and that is what I did* (Participant 7, male).

Interviewees highlighted the significance of sufficient sleep to preserve stable mental health, well-being, and daily functioning. While sleep quality varied between participants, most usually slept well. There were concerns about healthy sleep routines, but and some found it challenging to maintain these. One stated:

*There are nights when I do not sleep. I know what is vital to promote sleep, but it is one thing to know and another to actually do it. For example, I should not watch TV until bedtime, but I still do. I also should not use my iPad late at night, but do not you think I still sit and watch it before bedtime?* (Participant 2, female).

Several participants found physical activity to influence health and well-being positively. Specific activities varied, with one swimming 3000 m a week to stay in shape and another reporting they used to play handball but now enjoyed walking in nature and camping with his children. Many enjoyed walking alone, with friends, or with their dogs, and reported more walks during the summer than in the winter. A few participants were discontented with their inadequate physical health, which restricted them from engaging in physical activity, despite being aware of the antidepressant benefits of such activities.

### Theme 2: Social health determinants in coping with mental Illness

This theme comprises three categories: family and friends, engaging in meaningful hobbies and activities, and the influence of employment on mental health. These social determinants influenced participants’ ability to lead a stable and meaningful life and manage health challenges.

#### Category 6: Family and friends – a double-edged sword

Many participants talked about the importance of their family and friends in managing their daily lives and social participation. Friends and relatives provided support, encouragement, and motivation to help navigate life’s challenges and uncertainties. Several interviewees expressed that they enjoyed spending time with their parents, children, or grandchildren. These social relationships played a vital role in helping them deal with stress, find a safe space to discuss their problems, and share their successes.

For instance, one man lived alone but often felt lonely. Thus, he spent some nights at his parent’s home, receiving food and help with transportation as he did not own a car. Another noted he was grateful for his children, who lived at home with him. He enjoyed visiting cafes with his daughter and found this meaningful. He also provides support to a friend who struggled with his mental health. One female participant had a supportive network through her children, grandchildren, and helpful friends. She added:

*I have a very active social life, and I have always had many friends, many of them for different purposes. I always have someone I can visit and talk to* (Participant 2, female).

However, interviewees also experienced loss and complicated social relationships. One participant discussed how his relationships became complicated due to years of mental illness and substance use, resulting in limited contact with parents, children, and grandchildren. Another was struggling with the death of his spouse about a year ago after 30 years of marriage. However, he fondly reminisced about their shared adventures. Similarly, a woman struggled with losing her husband and the void it created. She particularly missed the ability to confide in him, as she valued his trustworthy nature. She expressed:

*One of the things I miss most about my husband is being able to talk to him and complain to him. I knew he would never tell anyone so I could tell him everything. My husband used to look after me, but now my children are trying to take that role instead* (Participant 2, female).

#### Category 7: Engaging in meaningful hobbies and activities

Interviewees highlighted the importance of hobbies as a way to capture their attention positively, bring enjoyment, and add meaning to their lives. Some enjoyed knitting, gaming, cooking, or having pets, while others mentioned surfing and sailing with family in the summertime. One interviewee had a sailboat that he had refurbished with his father and had invested much time in this practical activity. One woman mentioned having several hobbies and having trouble finding time for them all:

*I have so many hobbies I can engage in. I especially enjoy having birds. It is nice to watch and to feed them. I also enjoy knitting, beading, and sewing. I would like to read more. I have many books but do not always have time to read them* (Participant 4, female).

One individual used to have a personal support contact taking him out on activities. Personal support contacts help other people spend their free time actively and in a meaningful way. However, this offer was no longer available due to economic constraints in the municipality. Now he often spends time alone. Despite feeling overwhelmed, he tries to join in hikes and activities at a peer-driven café. One woman had experience singing in a choir and found joy in listening to music. Another has a professional from mental health home care who helps with transportation to social activities such as birthdays, seaside strolls, dining out, museum visits, and shopping.

#### Category 8: “Having a job has probably saved me” – the influence of employment on mental health

The interviewees’ diverse employment experiences highlight the positive and negative impacts of work on health. While employment can offer structure, purpose, secure income, and economic control, work-related stress can lead to deteriorated mental illness. Notably, participants found that being open about their mental health challenges with colleagues created a sense of understanding that promoted health and well-being.

Participants were employed in different fields, including engineering, information technology (IT), psychiatric care, grocery stores, and construction. For example, one person attended engineering school, worked in IT, and performed well in the same job for over two decades. Another person was currently employed at a psychiatric ward, and his experience as a patient provided valuable insights into the needs of patients in this setting. One interviewee worked as a painter and received help from colleagues due to his bad shoulder. This assistance enabled him to continue working.

Interview participants highlighted the importance of their work environments and colleagues for their health and well-being, noting how positive work experiences helped protect them from negative health consequences. One illustrated this:

*I have always been employed. Having a job has probably saved me. It would not be good for me to just sit at home staring at the wall. I saw a psychiatrist the last time I was worried about starting a new job. We briefly discussed it and altered my medication to help me relax, and everything went well from there* (Participant 8, male).

Some individuals experienced work-related stress that exacerbated mental illness symptoms, leading to instances of sick leave or disability benefits. One individual achieved greater stability by transitioning to a daytime position, contrasting a previous stressful job that required working during evenings, nights, and weekends. Another had tried several jobs, but work-related stress contributed to the deterioration of his mental illness. However, he later gained an education in IT. He said:

*It worked much better with education in adulthood rather than in high school. I enjoy working in the IT industry. I realized I fit in well and received positive feedback from colleagues and partners* (Participant 9, male).

However, interviewees may require sick leave during high stress and pressure at work. One gave an example:

*We had been under a lot of pressure for six months. Therefore, I had no choice but to take sick leave for over a week. I returned to work before fully recovering because my colleague was under more pressure and I needed to return quickly* (Participant 9, male).

## Discussion

In this qualitative study of individuals from the TIPS cohort diagnosed with SMI approximately 20 years ago, we identified key determinants of health perceived by these individuals as important for their well-being and ability to live a meaningful life. Our results revealed two themes covering eight categories of health determinants: access to mental healthcare adapted to individual needs, strategies during deterioration, use of psychotropic medication, maintenance of physical health, lifestyle, family and friends, engaging in meaningful hobbies and activities, and the influence of employment on mental health.

We found that access to mental healthcare and coordinated services that address individual needs was a crucial determinant. Coordinated care is essential for individuals with SMI to avoid falling through gaps in the system and achieve recovery [15]. Individuals with SMI valued professionals who were responsive to their needs, timely initial assessments, subsequent interventions, assistance navigating healthcare, and support for maintaining independence in the community [55]. We found that interviewees’ mental healthcare ranged from follow-up solely by GP to additional support from municipal mental healthcare and outpatient treatment from the CMHC. Our findings align with another Norwegian study [56], which detected similar variation in services provided to individuals with SMI. Notably, the highest level of patient satisfaction was reported for healthcare provided by GPs, highlighting the key role that GPs play in the care of individuals with SMI [56]. Access to supportive therapeutic environments is also vital [14]. Our findings reveal that longstanding therapeutic relationships with a known and trusted therapist positively affected health, well-being, and recovery. Other studies have similarly found that a mutual relationship of trust is imperative for sufficient mental healthcare [21, 27, 55].

Our study helps to illustrate the extreme difficulty of living with psychosis and the frustration experienced by individuals who are vulnerable to deterioration and have difficulties managing symptoms, particularly when faced with stressors. It is known that psychological distress and difficulties managing symptoms are common challenges for individuals with SMI [15]. Our findings underscore the importance of effective strategies to tackle mental illness deterioration, such as assistance in seeking mental healthcare, close follow-up of symptoms, use of emergency psychiatric care plans and acute teams, as well as emergency room contact and psychiatric admission. These strategies can prevent or minimize the adverse impact of deterioration on individuals’ health and well-being [20].

We identified psychotropic medication as an important determinant in preventing deterioration and maintaining stable health and well-being. Psychotropic medications are considered a cornerstone of SMI treatment [57] and are prescribed and used more commonly than other types of mental healthcare [58]. Even so, our findings show that side effects, such as fatigue, seizures, emotional flattening, and weight gain, can cause non-adherence. A systematic review [59] documented that while individuals with SMI are generally positive to psychotropic medication for short-term treatment, they are more skeptical towards using medication long-term use due to troublesome side effects. This view was evident among the interviewees in our study, who expressed a desire to reduce their dosage. Medication should be prescribed according to individual symptom severity, functioning, and experience [59]. This can, however, be challenging, as many struggle to communicate side effects to their doctors and, as such, may downplay or minimize their complaints [22]. Further, doctors do not always practice adequate serum medication level measurement, requiring individuals to remind their doctors when necessary [22]. Previous research [60, 61] has identified individual variations in how patients experience medication treatment. For example, fully recovered individuals considered antipsychotic medication a supplement to trustful patient-doctor relationships [62]. Professionals can enhance medication adherence by offering patients empathetic support throughout their illness, providing them with condition-specific information, and engaging them in treatment decision-making [60, 63].

Maintaining physical health emerged as a key health determinant. Problems with physical health negatively impacted interviewees’ well-being and activities of daily living. Individuals with SMI tend to have elevated risk of physical health problems and poorer clinical outcomes [10]. One explanation is low rates of examinations and underutilization of physical healthcare, which likely contribute to increased cardiovascular mortality in these individuals. As such, efforts are needed to prevent, detect, and treat physical illness in this group [64]. A related point is that physical symptoms can sometimes be identified as symptoms of mental illness. For one of our participants, it was thought that hyperthyroidism could have triggered manic episodes. Coordinated care that encompasses physical and mental health is crucial in addressing the needs of individuals with SMI [27, 63, 65]. Peer support can maximize the impact of care coordination [66]. By utilizing their experiences, peers can connect individuals with the appropriate resources and services and act as advocates during interprofessional meetings [66]. Additionally, peer support during the transition from psychiatric inpatient care to the home can reduce readmission [67]. Self-management training can also improve general health outcomes and help individuals manage their mental and physical health [19].

Another health determinant in our study was lifestyle, including healthy eating and avoiding tobacco. Unhealthy lifestyle practices such as poor diet and smoking contribute to poor physical health and premature mortality in individuals with SMI [68, 69]. Our interviewees found it beneficial to keep alcohol consumption moderate and avoid illicit drugs, an observation that aligns with a previous study [70] that linked substance use to increased risk of psychotic experiences. Our results also showed that adequate sleep is imperative for preserving stable mental health, well-being, and daily functioning. Insufficient sleep is associated with adverse health outcomes and impaired daily function, particularly among vulnerable populations [71]. Implementing measures that promote lifestyle modifications and self-care practices can further enhance health and well-being [63].

According to our results, family and friends were critical to achieve social participation and stable functioning. For individuals with SMI, social support is a key to living a meaningful life within the community [72]. Previous research has highlighted an inverse association between social network size, psychopathology, and illness symptoms in SMI [3]. Social network size does not consistently correlate with reported loneliness, indicating that the quality of relationships may be more important than quantity [21]. In addition, the frequency of interactions with friends positively predicts clinical recovery in psychosis over two years [73]. Our study showed that social relationships could be complicated due to years of mental illness and, in some cases, substance use, resulting in limited contact with family and friends. Such social fragmentation is associated with a higher prevalence of SMI [3], and loneliness and social isolation have been linked to poor health and reduced longevity [74].

Our study found meaningful hobbies and activities to be positive social health determinants. Engagement in meaningful activities is linked to positive emotional experiences, pleasure, and satisfaction with life [16]. Research supports [13, 14, 24] that social engagement promotes health by helping people rebuild their lives, facilitates social connections and recovery, and enables individuals to assert their needs. Activities such as taking care of other people or pets may provide subjective experiences of meaning, belonging, and connectedness, which, in turn, can improve health and quality of life [16].

Relatedly, employment was another important determinant. Being employed can offer structure, purpose, social interaction, chances to contribute to the community, and a secure income [16]. Holding a job can help individuals rebuild confidence, promote health, and foster a positive self-image [14]. That said, we identified a potential negative impact of work-related stress on health. Stress can exacerbate mental illness symptoms and lead to sick leave or disability insurance. Workplace factors such as working conditions, night shifts, job strain, psychosocial stressors, and unemployment could cause mental health to deteriorate [21]. It is common for individuals with SMIs to struggle to maintain employment [13, 15]. Stigma may further exacerbate any difficulties, hindering access to employment and perpetuating social inequalities [12].

### Limitations

Our study has limitations that should be acknowledged. First, the sample was drawn exclusively from a geographic area that practices early detection and intervention in first-episode psychosis, which is known to enhance health, well-being, and social function. Consequently, our findings may not capture the experiences of individuals with SMI living in other settings, potentially excluding important insights. Nevertheless, gaining knowledge on determinants of health from individuals who have been diagnosed with SMI for many years provides unique insights into the interconnectedness of health determinants and their long-term influence on health and well-being. Second, interviewees were selected based on their willingness to participate, introducing the possibility of a reporting bias if participants withheld information or gave strategic answers. Third, we did not use quantitative data, which could have enabled broader and complementary perspectives on health determinants. The transferability [51] of our findings may be limited due to the specific Norwegian context and the relatively small sample size. Nonetheless, we believe that the determinants of health identified here are relevant for similar international contexts.

## Conclusion

In this qualitative study, we explored determinants of health among individuals from the TIPS cohort diagnosed with SMI approximately 20 years ago. Our analysis identified mental, physical, and social determinants that enable and challenge the health and well-being of individuals with SMI and their capacity to live a meaningful life. Study results contribute to the early intervention in the psychosis research literature by offering qualitative insights into important health determinants, the specific long-term needs of individuals with SMI, and the importance of long-term coordinated care. Our results have implications for clinical practice, highlighting the need for individually tailored and coordinated care that comprehensively addresses these determinants. Promoting engagement in meaningful activities and facilitating employment opportunities can enhance health in individuals with SMI, warranting further research into relevant interventions. There is also a need to investigate ways to maintain physical health and optimize individualized medication management.

## Data Availability

The datasets analyzed during the current study will be made available to appropriate academic parties upon reasonable request to the corresponding author.

## List of abbreviations

CMHC: Community mental health center
REK: Regional Committee for Medical and Health Research Ethics
SMI: Serious mental illness
WAIS-R: Wechsler Adult Intelligence Scale Revised
